# Macrophage membrane camouflaged reactive oxygen species responsive nanomedicine for efficiently inhibiting the vascular intimal hyperplasia

**DOI:** 10.1186/s12951-021-01119-5

**Published:** 2021-11-17

**Authors:** Boyan Liu, Wenhua Yan, Li Luo, Shuai Wu, Yi Wang, Yuan Zhong, Dan Tang, Ali Maruf, Meng Yan, Kun Zhang, Xian Qin, Kai Qu, Wei Wu, Guixue Wang

**Affiliations:** 1 Key Laboratory for Biorheological Science and Technology of Ministry of Education, State and Local Joint Engineering Laboratory for Vascular Implants, Bioengineering College of Chongqing University, Chongqing, 400044 People’s Republic of China; 2 The Second Affiliated Hospital of Chongqing Medical University, Chongqing, 400010 People’s Republic of China

**Keywords:** Intimal hyperplasia, Nanomedicine, Targeted delivery, Macrophages, ROS-responsive

## Abstract

**Background:**

Intimal hyperplasia caused by vascular injury is an important pathological process of many vascular diseases, especially occlusive vascular disease. In recent years, Nano-drug delivery system has attracted a wide attention as a novel treatment strategy, but there are still some challenges such as high clearance rate and insufficient targeting.

**Results:**

In this study, we report a biomimetic ROS-responsive MM@PCM/RAP nanoparticle coated with macrophage membrane. The macrophage membrane with the innate “homing” capacity can superiorly regulate the recruitment of MM@PCM/RAP to inflammatory lesion to enhance target efficacy, and can also disguise MM@PCM/RAP nanoparticle as the autologous cell to avoid clearance by the immune system. In addition, MM@PCM/RAP can effectively improve the solubility of rapamycin and respond to the high concentration level of ROS accumulated in pathological lesion for controlling local cargo release, thereby increasing drug availability and reducing toxic side effects.

**Conclusions:**

Our findings validate that the rational design, biomimetic nanoparticles MM@PCM/RAP, can effectively inhibit the pathological process of intimal injury with excellent biocompatibility.

**Graphical Abstract:**

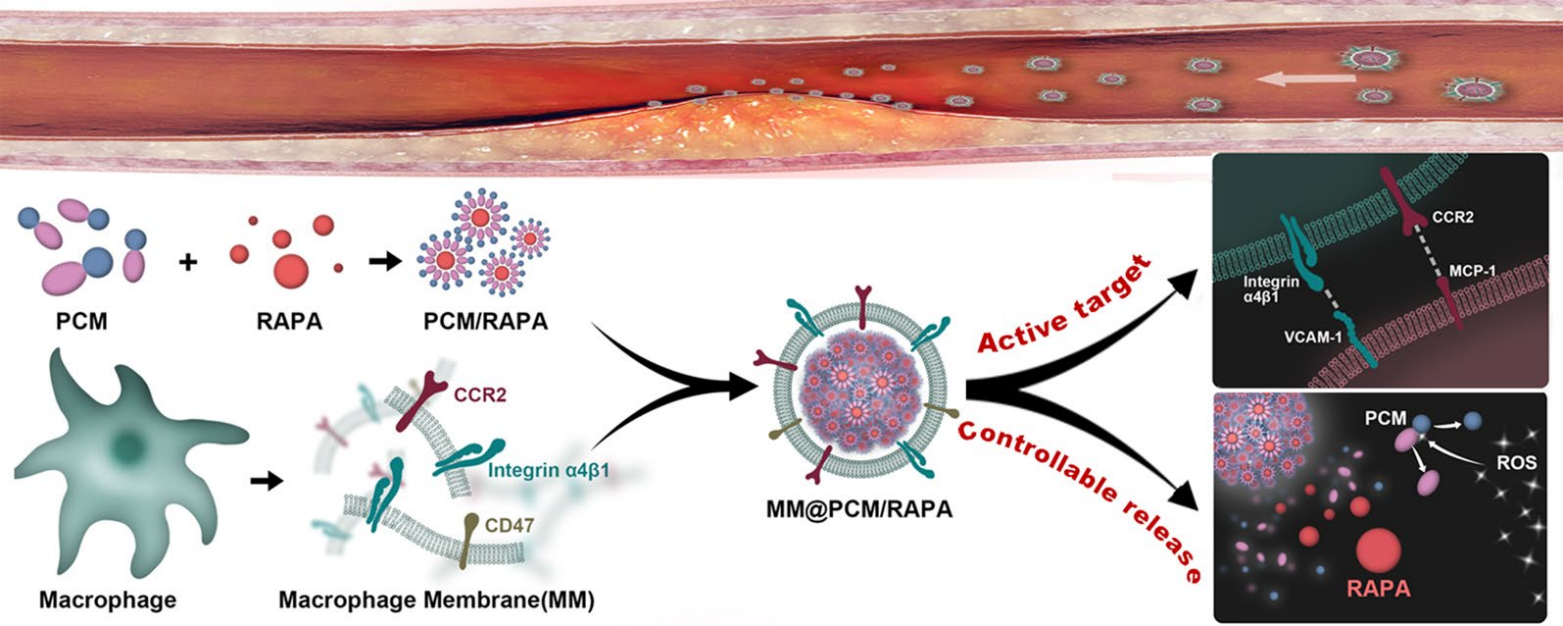

**Supplementary Information:**

The online version contains supplementary material available at 10.1186/s12951-021-01119-5.

## Introduction

Intimal hyperplasia (IH) is the main cause of stenosis or occlusion after the injured vascular reconstruction and endovascular therapy, considering as the main pathological basis of vascular diseases [1, 2]. In clinic therapy, the long-term dosing in early stage has low absolute bioavailability and undesirable side effects [3]. For serious pathology, percutaneous stent implantation is widely used to restore the blood flow [4], but subsequent in-stent thrombosis and in-stent restenosis become an unforeseen fatal risk [5, 6]. Recently, nano-drug delivery system has receive considerable attentions as a noninvasive therapeutic platform for the most diseases because of the attractive performances in medicament improvement, such as solubilizing and stabilizing drug delivery, enhancing therapeutic bioavailability by the target drug delivery and the controllable drug release, and significantly reducing drug toxicity and side effects [7–10].

In the harsh physiological environment in vivo, the traditional nanocarrier is hard to be approved by the mononuclear phagocyte system, and inevitably be cleared as the “foreign” by organism [11], which will result in massive drug losses and unfavorable side effects. To address this challenge, the biomimetic cell membrane camouflaging strategy has been engineered into the biomimetic nano-drug delivery system for developing the long-term blood circulation and the innate biological target function [12, 13], especially the innovative applications in cardiovascular diseases (Additional file 1: Table S1). Zhang et al. firstly reported a natural red blood cell membrane encapsulated poly(lactic-*co*-glycolic acid) (PLGA) nanoparticle to significantly prolong the circulation time in vivo compared with the naked PLGA and the PEGylated PLGA [14]. In our previous reports, RBC camouflaged PLGA loading with the rapamycin (RAP) has developed to enhance the target therapy efficacy in atherosclerosis at first [15]. Recently, many other types of cell membranes, such as macrophage, platelet, white blood cell, cancer cell, stem cell [11] have been involved to develop the biomimetic nanomedicine. For example, Dehaini et al. hybridized erythrocyte membrane and platelet membrane to endow nanocarriers as the advanced theranostics with the long-term blood circulation and the active target ability to lesion [16]. Cheng et al. fabricated a macrophage membrane coated nanoparticle for atorvastatin delivery as the efficient therapeutic agent for atherosclerosis [17]. Because of the innate ability to perceive, integrate and respond to the dynamic environment in vivo, the biomimetic nanocarrier based on cells and cell membrane engineered nanomedicines are capable of inheriting the innate functions of immune escape and target immigration for promoting drug target delivery to lesion.

Neutrophils and macrophages are the most attractive candidates for the specific biological efficacy, i.e. as the main cellular components of the innate immune response, which can be superiorly recruited and accumulated into the inflammation site [18]. In our previous reports, the biomimetic nanocarriers camouflaged with the macrophage membrane is capable of inheriting the innate “homing” capacity to selectively accumulate at the atherosclerotic pathology [19, 20]. In fact, during the vascular injury pathology in IH, the over-expressed cytokines and chemokines (such as interleukin, tumor necrosis factor-α, monocyte chemoattractant protein-1) will active the endothelial cells (ECs) with the high-expressed vascular cell adhesion molecule-1 (VCAM-1), which is able to regulate the inflammatory cells (neutrophils and monocytes) immigration and accumulation into the injured lesion through the specific recognition of integrin α4β1 on surface resulting in the active target delivery to the IH [21–25].

Besides the target cargo delivery, smart nanomedicines have been widely improved to respond to the endogenous-exogenous stimuli (such as light, magnetic field, pH, etc.) for “on-demand” drug release in the pathological lesion [26]. Especially, the endogenous pathological stimulation is widely proved to be a fantastic and feasible strategy to trigger the local cargo release for enhancing the therapeutic efficacy, as well as reducing the undesirable side effects [27]. It is well known that ROS is usually over production in vascular dysfunction, and accelerates the pathological deterioration [28–30]. Wei et al. developed a bioengineered ROS-responsive nanoparticles to protect against ischemic brain damage [31]. The results show that the ROS-responsive nanoparticles can significantly enhance the active targeting ability, and improve the neurological score and infarct volume after treating the middle cerebral artery occlusion (MCAO) mice model. Therefore, the smart nanocarrier in response of the endogenous pathological ROS stimulus could be a feasible platform to improve the “on-demand” drug release for the safe and efficient therapy in IH.

**Fig. 1:**
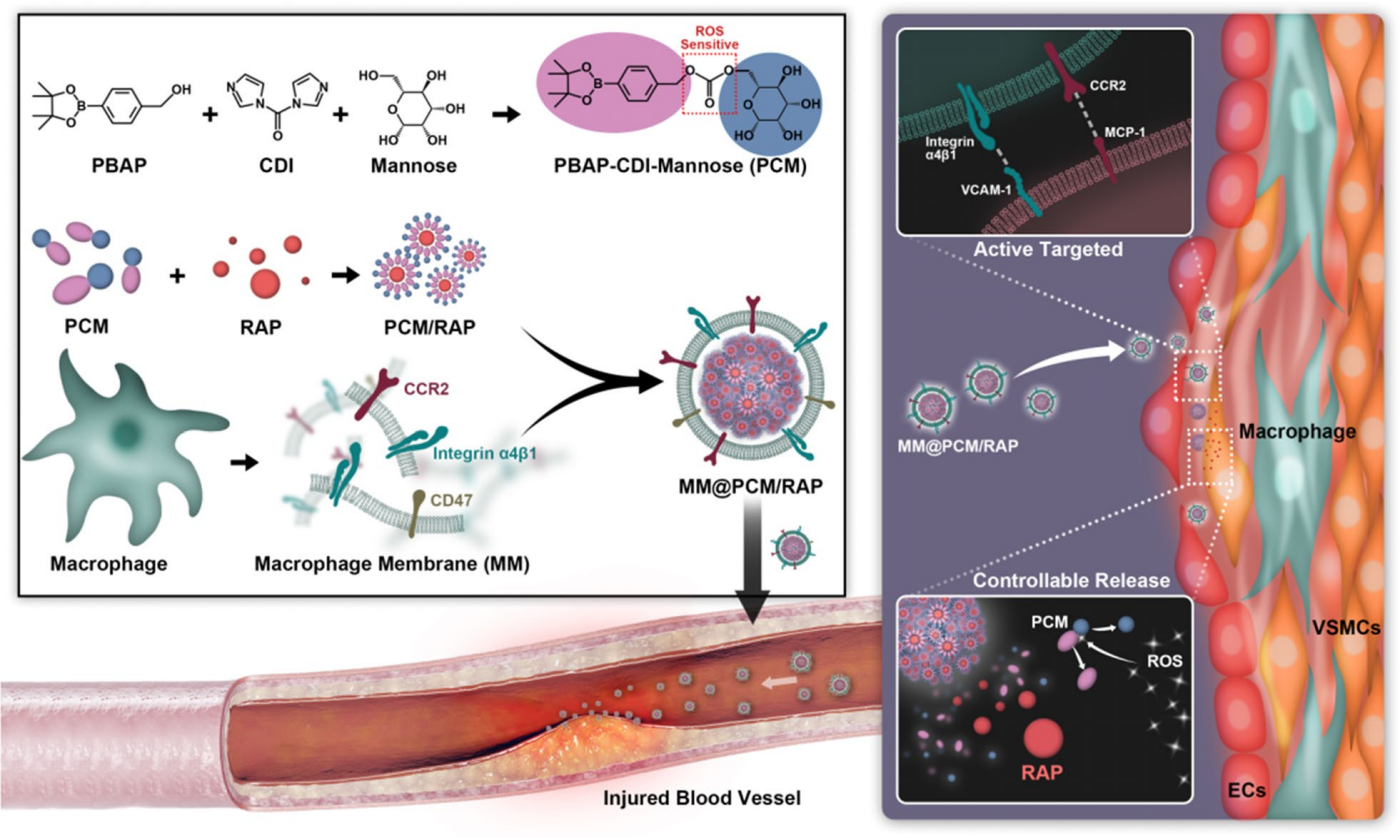
Illustrations of MM@PCM/RAP for the treatment of IH

Herein, in this study, the biomimetic smart nanomedicine (MM@PCM/RAP) was constructed for target therapy in IH. The amphipathic low-molecule self-assembly (PCM) was firstly synthesized from the PBAP functionalized *D-*mannose, and subsequently was coated using macrophage membranes (MM), which endowed nanomedicine the biomimetic abilities, such as escaping the clearance by immune, selectively delivering to lesion. Moreover, the amphipathic carrier PCM can effectively solubilize the model drug RAP, and timely respond to the high concentration level of ROS in pathological environment for triggering the local drug release (Fig. 1). Therefore, the biomimetic smart nanomedicine MM@PCM/RAP would be a promising and feasible candidate as the safe and efficient therapeutic nanoplatform for IH.

## Results and discussion

### Preparation and characterization of MM@PCM/RAP

The ROS-responsive amphiphilic molecule (PBAP-CDI-Mannose, PCM) was synthesized by conjugating 4-(hydroxymethyl) phenylboronic acid pinacol ester (PBAP) with *D*-mannose. the chemical structure of PCM was confirmed by ^1^H NMR (Additional file 1: Fig. S1). To improve the encapsulation efficiency of RAP in aqueous solution, PCM/RAP self-assemblies were fabricated through dialysis method [32]. Due to the amphiphilic property, *i.e.* the hydrophobicity of PBAP and the hydrophilicity of mannose, PCM could self-assemble by encapsulating RAP into the hydrophobic core in aqueous solution for harvesting the drug loaded nanoparticles (PCM/RAP). According to the UV–Vis measurement, the drug encapsulation efficiency (*EE*) and drug loading efficiency (*LE*) of PCM/RAP were 79.7 ± 0.2% and 7.3 ± 0.5%, respectively (Additional file 1: Table S2). In the PCM/RAP solution, the solubility of RAP was significantly improved (≈ 159.4 μg mL^−1^), which could significantly solubilize the hydrophobic drug RAP as over 60 times higher than its solubility (≈ 2.6 μg mL^−1^) in aqueous solution. Due to the ROS-sensitivity of PBAP moieties on PCM, the PCM/RAP nanoparticles would disassemble when it exposed to high concentration level of ROS environment. Visually, the blue visual effect (Tyndall effect) would gradually fade along with the PCM/RAP nanoparticle degradation. As shown in Figure S2, the blue color of co-incubated solution (PCM/RAP with 1 mM H_2_O_2_) was obviously faded at 1 h, and was nearly colorless at 24 h, while no significant color change was observed in the solution without H_2_O_2_ stimulus.

**Fig. 2:**
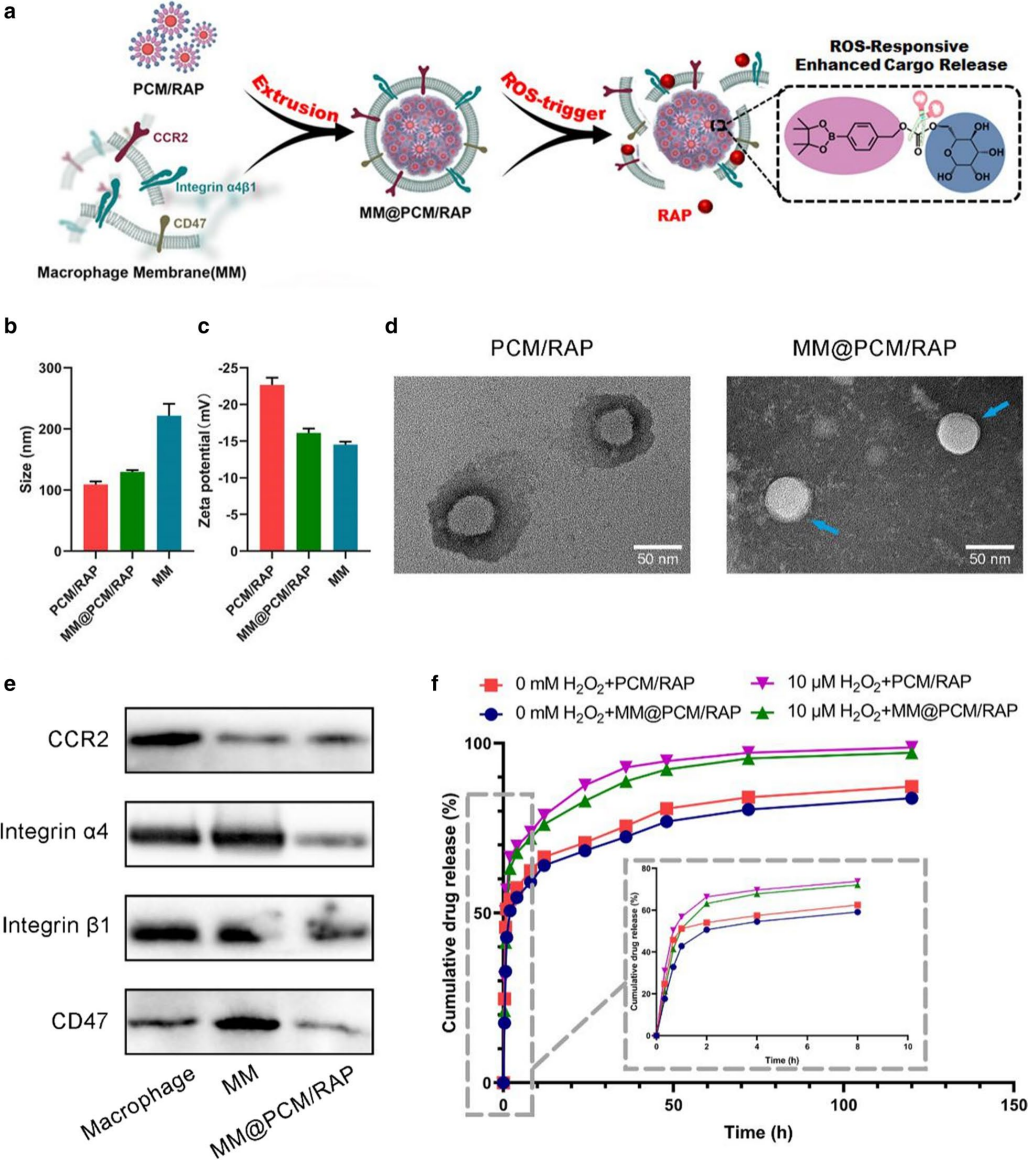
Characterization of PCM/RAP and MM@PCM/RAP. **a** Schematic illustration of preparation and drug release of MM@PCM/RAP. **b** The hydrated particle size and **c** zeta potential of PCM/RAP, MM@PCM/RAP and MM (*n* = 3, mean ± SD). **d** TEM images of PCM/RAP and MM@PCM/RAP (scale bar = 50 nm). **e** Western blot analysis of CCR2, Integrin α4, Integrin β1 and CD47 in Macrophage, MM and MM@PCM/RAP. **f** In vitro drug release study of PCM/RAP and MM@PCM/RAP without and with 10 μM H_2_O_2_ (*n* = 3, mean ± SD)

To harvest the biomimetic ROS-responsive MM@PCM/RAP nanoparticles (Fig. 2a), the MM was first isolated from murine macrophage RAW 264.7 cell line, and was subsequently coated onto the surface of PCM/RAP nanoparticles via an extrusion method [33]. Dynamic laser scattering (DLS) was employed to determine the hydrodynamic diameter and zeta potential of PCM/RAP and MM@PCM/RAP nanoparticles. As shown in Fig. 2b, after coated with membrane, the average hydrodynamic diameter of nanoparticles increased about 20 nm (from 109.4 to 129.6 nm), which might be ascribed to thickness of the functionalized MM on surface [34, 35]. The zeta potential decreased to a similar level to macrophage membrane, which revealed successful fabrication of MM@PCM/RAP (Fig. 2c). Furthermore, the negative charge surface was beneficial for inhibiting the protein absorption to form protein corona on surface by the electrostatic repulsion effects (Additional file 1: Fig. S3), which is important to maintain the physicochemical properties and the biological performance of nanomedicine in vivo. Subsequently, the core–shell structure of the MM@PCM/RAP nanoparticles was further visibly confirmed by TEM investigation and cell phagocytosis co-localization experiment. As the TEM results shown in Fig. 2d, compared with PCM/RAP nanoparticles with homogenous spherical nanoscale morphology, a corona layer was observed on the surface of nanoparticle. and. After co-incubation for 4 h using the fluorescent labeled nanoparticles (the red fluorescent DiD replacing RAP to stain the nanoparticle, and the green fluorescent DiO stained cell membrane), the phagocytosis of fluorescent nanoparticles by ECs was observed by CLSM. As shown in Figure S4, the red region near the ECs highly overlapped with the green region, which revealed that the macrophage membrane was successfully coated on the nanoparticles.

Except the MM coating structure in macroscopy, SDS-PAGE analysis was used to determine retention of the protein composition on surface. The result showed that MM@PCM/RAP nanomedicine was able to inherit almost of all proteins compared with the original macrophage membrane (Additional file 1: Fig. S5). Furthermore, the expression of key proteins for target function on macrophage membrane were detected by western blot (WB), such as CCR2 (a receptor for monocyte chemoattractant protein-1 (MCP-1)), integrin α4β1 (receptors for vascular cell adhesion molecule-1 (VCAM-1)) and CD47. As previous reports [36, 37], CCR2 and integrin α4β1 proteins could promote the recruitment of monocytes to “home” the inflammatory lesion, which provided the vital basis for improving MM@PCM/RAP to enhance the active target delivery into lesion. Furthermore, CD47 could act function of “do not eat me” to prevent the undesirable phagocytosis by inhibiting binding to receptor SIRP-α on mononuclear phagocytes [38, 39]. The WB result showed that both CCR2, integrin α4β1 and CD47 were well retained on the surface of MM@PCM/RAP (Fig. 2e), which suggested that the biomimetic nanoparticles had potential to inherit the “homing” function of macrophage to escape immune clearance and selectively deliver to inflammatory lesion.

To investigate the ROS-responsive drug release profiles, RAP release from MM@PCM/RAP and PCM/RAP nanoparticles was studied in the PBS with or without H_2_O_2_ (10 μM) measured by UV–Vis (Additional file 1: Figs. S6 and S7). As shown in Fig. 2f, the drug release showed a rapid release in the first 2 h, then exhibited a steady slow release, and finally reached a plateau after 48 h. In response to H_2_O_2_, the accumulative drug release ratio of MM@PCM/RAP significantly increased from ≈ 59.1% to ≈ 72.0% in PBS without or with H_2_O_2_. The loaded RAP indeed was rapidly released from PCM, which should be resulted from the ROS-responsive degradation of carrier and the unstable cell membrane under the stimulus in H_2_O_2_. Interestingly, it was observed that the cumulative release of RAP in the MM@PCM/RAP sample was slightly lower than that in the PCM/RAP sample, which might be caused by the slight hindrance of the cell membrane on surface.

### Study on immune escape and active targeting

**Fig. 3:**
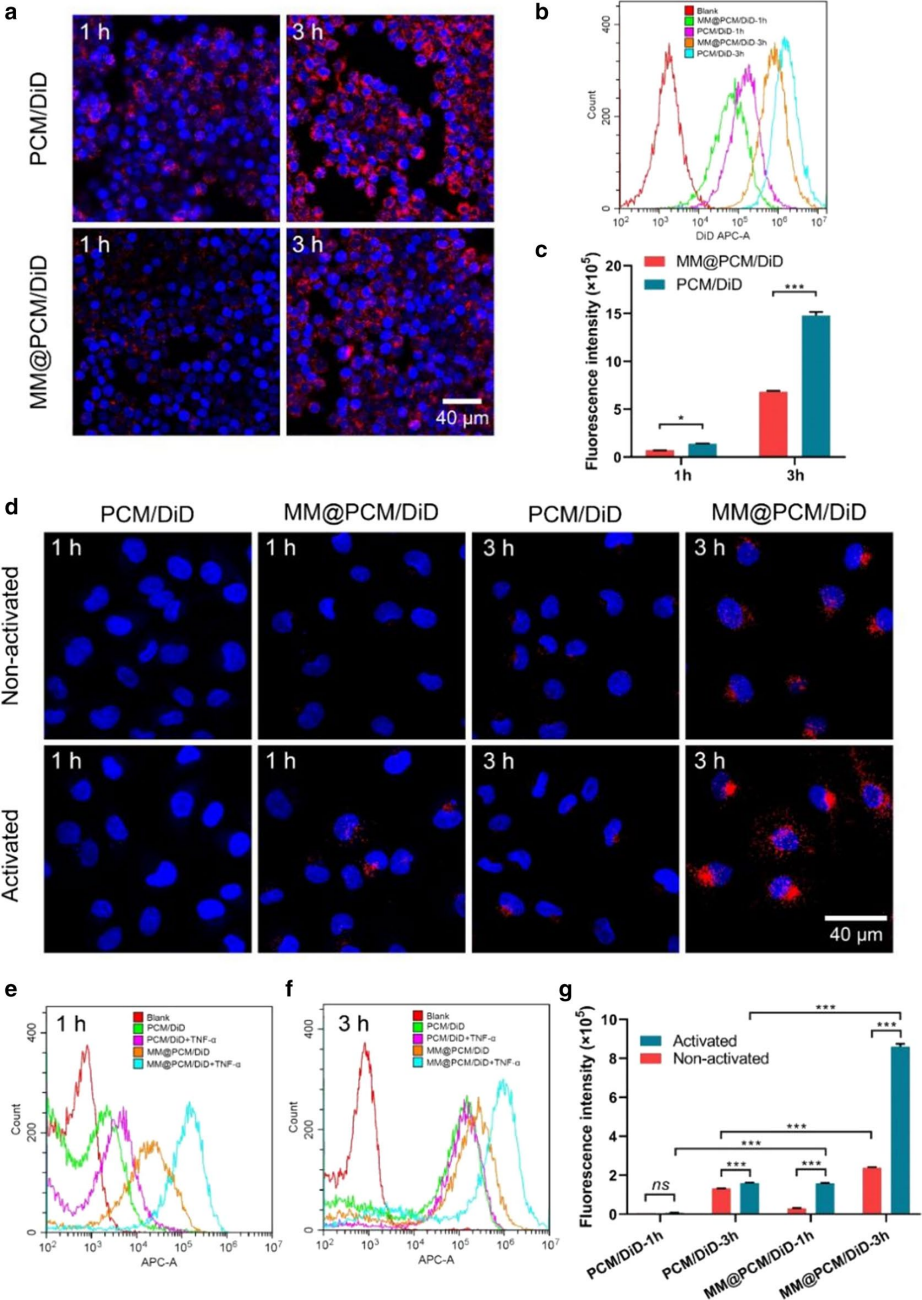
In vitro cellular uptake of PCM/DiD and MM@PCM/DiD by RAW 264.7 macrophages and ECs. Nuclei were stained with DAPI (blue), whereas nanoparticles were stained with DiD (red). **a** CLSM images of PCM/DiD and MM@PCM/DiD phagocytosed by RAW 264.7 cells at 1 h or 3 h (scale bar = 40 µm). **b** FACS images and **c** quantification anilysis of PCM/DiD and MM@PCM/DiD phagocytosed by RAW 264.7 cells at 1 h or 3 h (*n* = 3, mean ± SD). **d** CLSM images of PCM/DiD and MM@PCM/DiD cellular uptake by ECs without or with TNF-α activated at 1 h or 3 h (scale bar = 40 µm). **e**, **f** FACS images of PCM/DiD and MM@PCM/DiD cellular uptake by ECs without or with TNF-α activated at 1 h or 3 h. **g** Quantification of cellular uptake of PCM/DiD and MM@PCM/DiD in ECs without or with TNF-α activated at 1 h or 3 h (*n* = 3, mean ± SD). Significances among the groups were determined by one-way ANOVA, followed by post hoc pairwise comparisons with the Tukey or Dunn honest significant difference. *p < 0.05, **p < 0.01, and ***p < 0.001. *ns* no significance

After coated with macrophage membrane having CD47 functional protein on surface, the biomimetic nanoparticles should act autologous camouflage, thus reducing the clearance by mononuclear phagocyte system. The immune evasion function of the biomimetic nanoparticles was verified by the in vitro macrophage phagocytosis experiment. Here, for fluorescent tracing, RAP was replaced with red DiD to fabricate MM@PCM/DiD and PCM/DiD nanoparticles. After co-incubation with the murine macrophages RAW 264.7 for 1 h or 3 h respectively, the fluorescence intensity of macrophages phagocytosing nanoparticles was observed under confocal laser scanning microscopy (CLSM) and quantified by fluorescence-activated cell sorting (FACS). As shown in Fig. 3a and b, the phagocytosis of nanoparticles by macrophages showed a time-dependent cumulative effect, and the fluorescence intensity of the PCM/DiD group was significantly stronger than that of the MM@PCM/DiD group. According to FACS calculations, the fluorescence intensity of the PCM/DiD group was about twice than that of the MM@PCM/DiD group after 3 h incubation (Fig. 3c), indicating that the macrophage membrane coating strategy may be beneficial for nanoparticles escaping the clearance by macrophages.

As reported in previous studies [40], endothelial cells in the pathological lesion were an inflammatory, and expressed a series of chemokines and adhesion molecules, which facilitated the recruitment of monocytes at the pathological lesion. Consequently, the biomimetic nanoparticles functionalized with macrophage membrane were able to actively target to the pathological location. To verify the hypothesis, the activated ECs (treated with 40 ng mL^−1^ TNF-α for 24 h) and normal ECs were used for investigating the active targeting behavior for the inflammatory ECs. As shown in Fig. 3d–g, after co-incubating with MM@PCM/DiD for 3 h, the activated ECs showed strong red fluorescence, and the fluorescence intensity was 3.5 times than that of the non-activated ECs, indicating the macrophage membrane coating strategy could endow the nanoparticles with the active target ability to the inflammatory lesion.

**Fig. 4:**
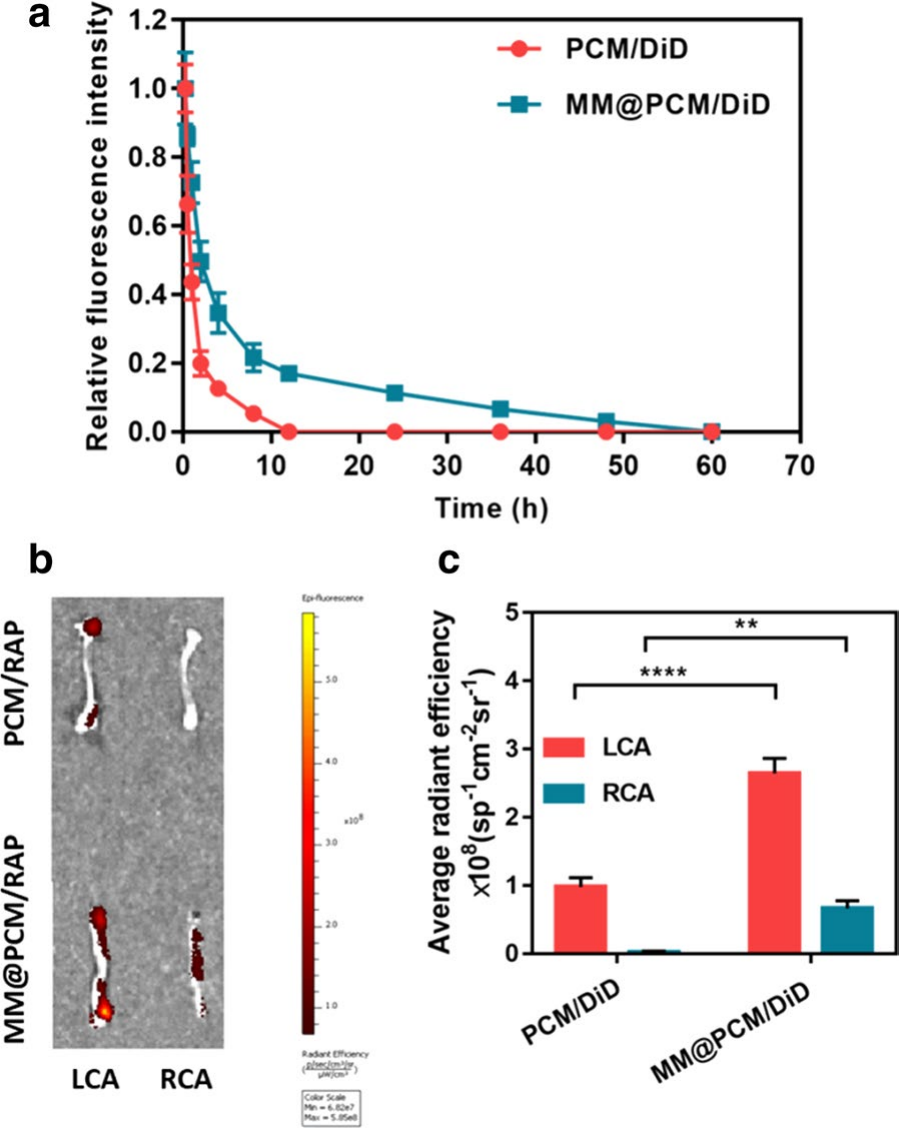
**a** Pharmacokinetic studies of MM@PCM/DiD and PCM/DiD, and **b** the ex vivo fluorescence image and **c** quantitative data of carotid artery (LCA: left carotid artery; RCA: right carotid artery) in mice (*n* = 5). **p < 0.01, and ****p < 0.0001

Moreover, the pharmacokinetic studies also confirmed that the biomimetic nanomedicine MM@PCM/RAP could significantly prolong the blood circulation time more than 5 times compared with the PCM/RAP (Fig. 4a). In addition, the ex vivo results confirmed that the biomimetic nanomedicine MM@PCM/RAP could selectively deliver and retained into the injured LCA, exhibiting the enhanced target efficacy (Fig. 4b and c).

### In vitro VSMCs inhibition

**Fig. 5:**
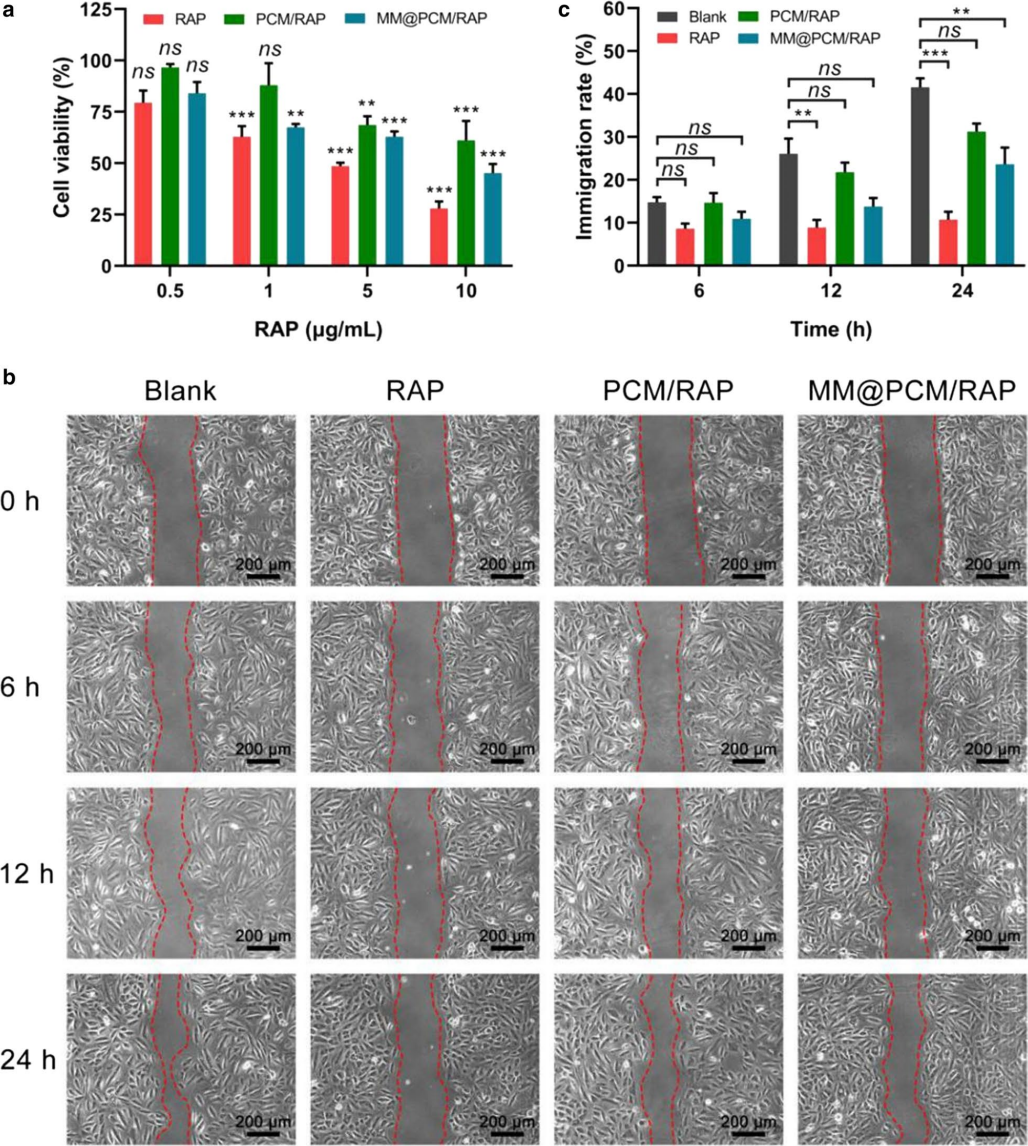
In vitro VSMCs inhibition. **a** Viability of VSMCs treated with free RAP, PCM/RAP, and MM@PCM/RAP, respectively, at 0.5, 1, 5 and 10 μg/mL RAP (*n* = 5, mean ± SD). **b** Photographs and **c** quantification analysis of VSMCs migration treated with free RAP, PCM/RAP, and MM@PCM/RAP, respectively, at 2.5 μg/mL RAP (scale bar = 200 µm, *n* = 5, mean ± SD). Significances among the groups were determined by one-way ANOVA, followed by post hoc pairwise comparisons with the Tukey or Dunn honest significant difference. **p < 0.01, and ***p < 0.001. *ns*, no significance

The proliferation and migration of vascular smooth muscle cells (VSMCs) is the main cause of IH. To study the inhibitory effect of biomimetic nanoparticles on the proliferation of VSMCs, VSMCs were treated with free RAP, PCM/RAP and MM@PCM/RAP with equivalent drug concentration (0.5, 1, 5, 10 μg mL^−1^) for 24 h, respectively, and the viability of VSMCs was determined by MTS assay. As shown in Fig. 5a, the inhibition of VSMCs showed a dose-dependent effect. When the concentration of RAP was 10 μg mL^−1^, the cell activity of VSMCs in free RAP, PCM/RAP and MM@PCM/RAP groups was 28.0%, 61.1% and 45.1%, respectively, which was significantly different from that in Blank group. The strong inhibitory effect of free RAP on VSMCs may be due to the direct drug effect without the release process, which accelerated the cell–drug interaction procedure. However, in vivo application, low solubility of free RAP would greatly limit the dosage, and free RAP would be efficiently eliminated by the immune system because of exogenous. Therefore, MM@PCM/RAP with the capability of immune escape, might have superior advantages in inhibiting the proliferation of VSMCs in vivo.

Furthermore, in vitro scratch assay was used to confirm the inhibitory effects on VSMCs migration. After the cell density reached about 100%, the monolayer of VSMCs were preformed uniform scratch wounds followed by incubating with the free RAP, PCM/RAM, MM@PCM/RAP and fresh medium (Blank), respectively. The RAP concentration was set to 2.5 μg mL^−1^ and the incubation medium used 0.2% FBS to reduce errors caused by cell proliferation. The recovery of the scratch gap was then recorded under a microscope at 0, 6, 12, and 24 h. The images were analyzed by Image J to quantify the mobility of VSMCs. As shown in Fig. 5b and c, after co-incubation for 24 h, the VSMCs migration rate of RAP group (10.7%) and MM@PCM/RAP group (23.6%) was significantly different from that of the blank group (40.9%). This results confirmed that MM@PCM/RAP might have a significant ultimate inhibitory effect on the proliferation and migration of VSMCs, which could be a desirable candidate for IH treatment in vivo.

### In vitro biosafety assessment

**Fig. 6:**
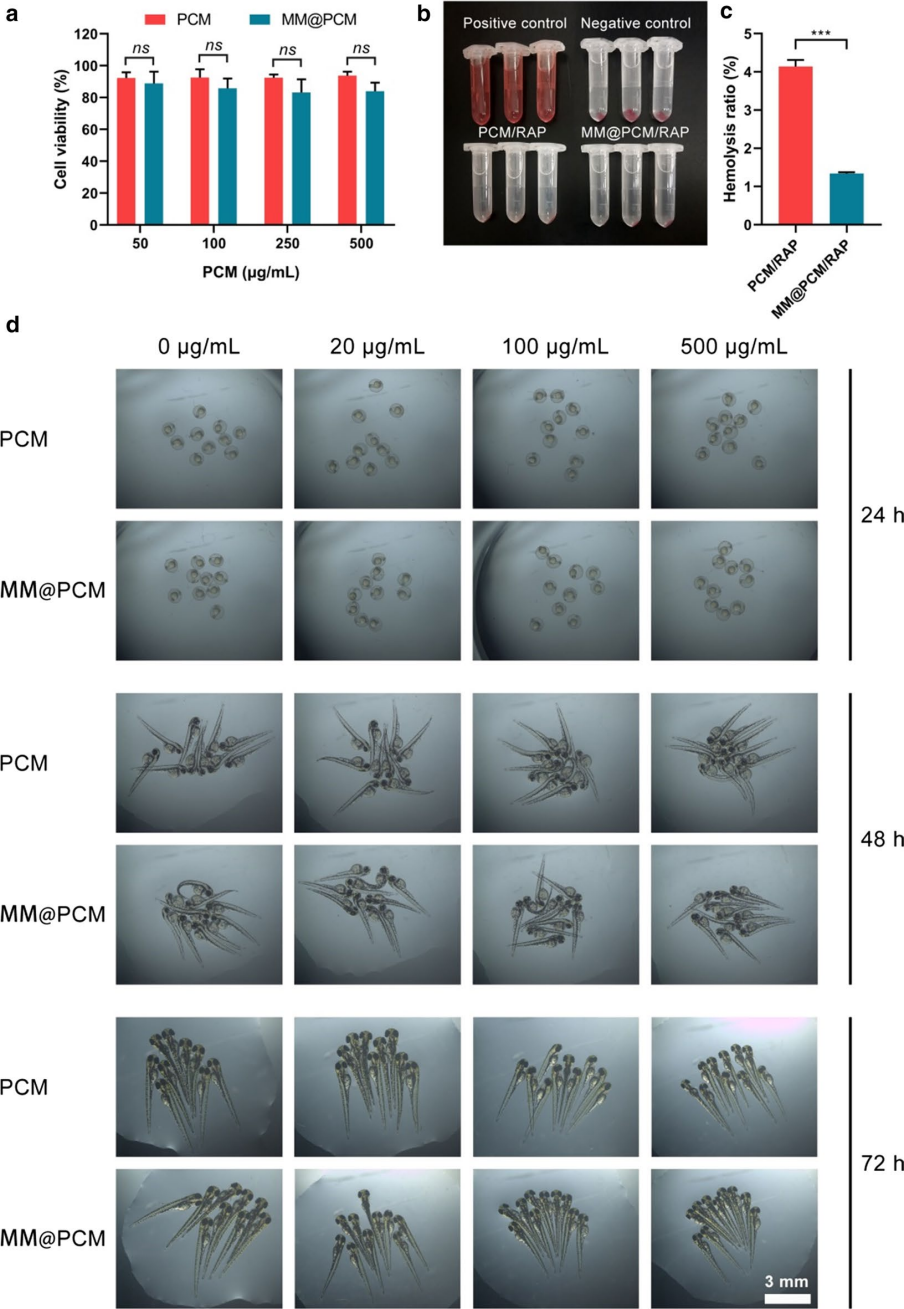
In vitro biosafety evaluation. **a** Endothelial toxicity of carrier PCM and MM@PCM (*n* = 5, mean ± SD). **b** The images and **c** quantification analysis of the hemolysis assay of PCM/RAP and MM@PCM/RAP (*n* = 3, mean ± SD). **d** Toxic effects of different concentrations of PCM and MM@PCM on zebrafish embryos (scale bar = 3 mm). Significances among the groups were determined by one-way ANOVA, followed by post hoc pairwise comparisons with the Tukey or Dunn honest significant difference. ***p < 0.001. *ns* no significance

The in vitro biocompatibility of the low-molecule self-assembly PCM and MM@PCM was verified by endothelial toxicity assay. For the potential application in vivo to early interact with the vascular endothelium, EC was selected as the model cell to verify the cell compatibility of carrier by MTS assay. After co-incubating with PCM and MM@PCM solution for 48 h, the cell viability of ECs was calculated. As shown in Fig. 6a, both PCM and MM@PCM had a favorable cell viability higher than 80%, even the concentration up to 500 μg mL^−1^, which indicated PCM and MM@PCM were no significant cytotoxicity. Subsequently, the blood compatibility of the nanoparticles was verified by hemolysis assay. As shown in the Fig. 6b and c, the hemolysis ratio of PCM/RAP and MM@PCM/RAP were 4.1% and 1.3%, indicating good blood compatibility. Moreover, zebrafish embryo assay was further conducted to verity the potential developmental toxicity. As shown in Fig. 6d, the images showed that the hatching rate of zebrafish embryos in all groups reached 100% and the zebrafish juveniles had no serious deformities, demonstrating that PCM and MM@PCM had desirable biocompatibility with no significant toxicity for potential application in vivo.

### Therapeutic efficacy on mice with carotid artery injury

**Fig. 7:**
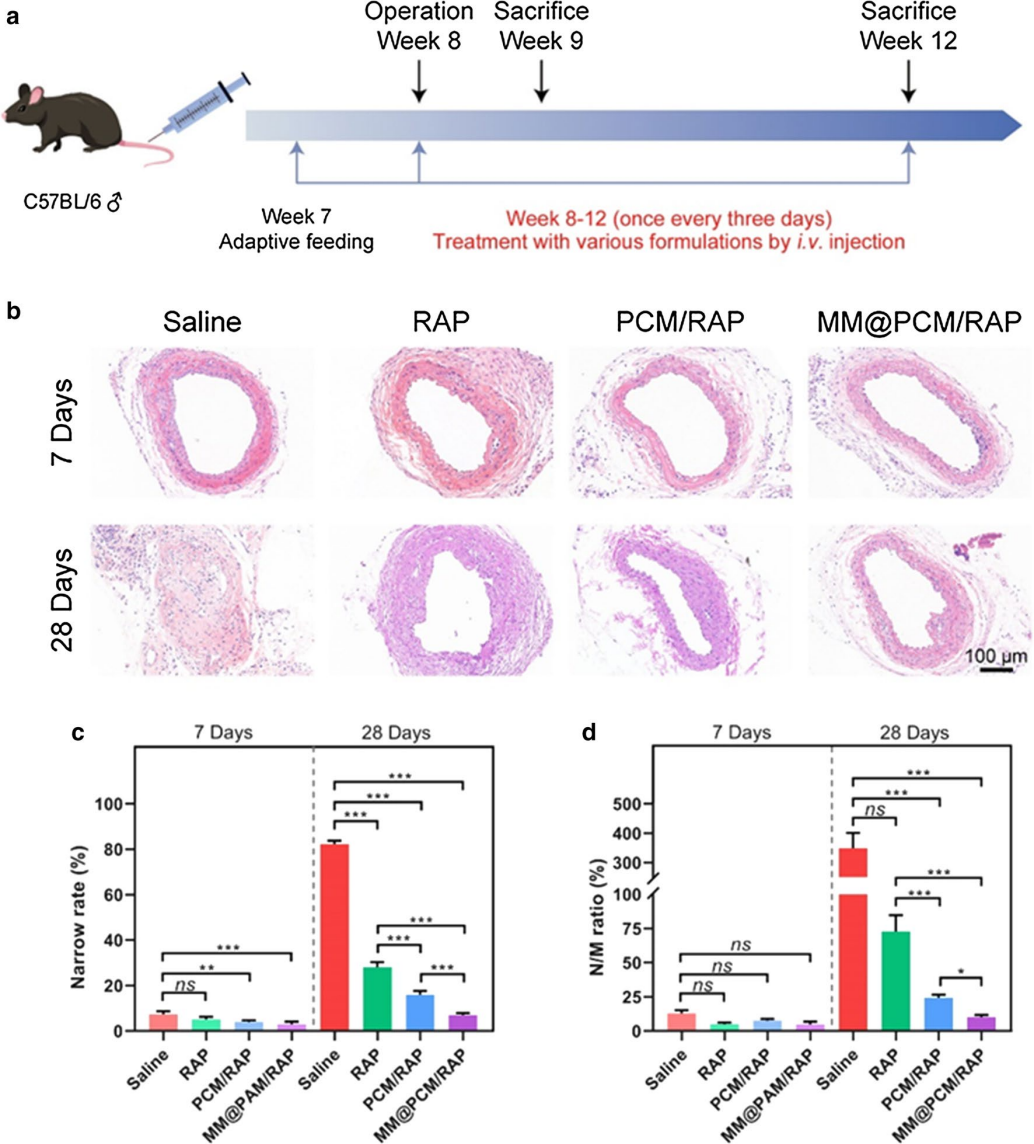
Therapeutic effects of *i.v.*-delivered MM@PCM/RAP in a carotid artery wire injury mouse model. **a** Schematic illustration of the treatment protocols. **b** H&E staining of carotid artery sections from mouse model after different treatments for 7 and 28 days. **c** Narrow rate and **d** N/M ratio of carotid artery sections (*n* = 6, mean ± SD). Significances among the groups were determined by one-way ANOVA, followed by post hoc pairwise comparisons with the Tukey or Dunn honest significant difference. *p < 0.05, **p < 0.01, and ***p < 0.001. *ns*, no significance

To study the inhibitory effect of biomimetic nanoparticles on IH, 8-week-old male C57BL/6 mice were used to construct a carotid artery wire injury model as the method described previously [41]. Then, the mice were treated with saline, free RAP, PCM/RAP and MM@PCM/RAP via tail vein injection, respectively. The first injection was performed within 10 min after the operation, and then the injection was continuously applied at a frequency of once every 3 days. The mice were sacrificed on 7 days and 28 days, respectively. Paraffin sections of mouse carotid arteries were prepared for subsequent experiments (Fig. 7a).

H&E staining was used to verify the tissue morphology of the injury (Fig. 7b–d). After treatment for 7 days, the obvious IH was observed in the saline group, while almost no hyperplasia occurred in the other three RAP-treated groups. Moreover, severe IH, even completely sealing the blood vessel, was observed in the saline group after treatment for 28 days, while the other three treatment groups showed an inhibitory effect. The stenosis rate of free RAP, PCM/RAP and MM@PCM/RAP group on 28 days was 28.1%, 15.9% and 6.9% respectively, which was significantly lower than that of saline group (82.3%), indicating that the biomimetic nanoparticles MM@PCM/RAP could significantly inhibit IH, which was extremely important to maintain blood flow homeostasis during the self-repair of the injured blood vessel. In addition, the decrease of the stenosis rate of MM@PCM/RAP compared with PCM/RAP may be due to the biomimetic target delivery contributed by the macrophage membrane on surface.

**Fig. 8:**
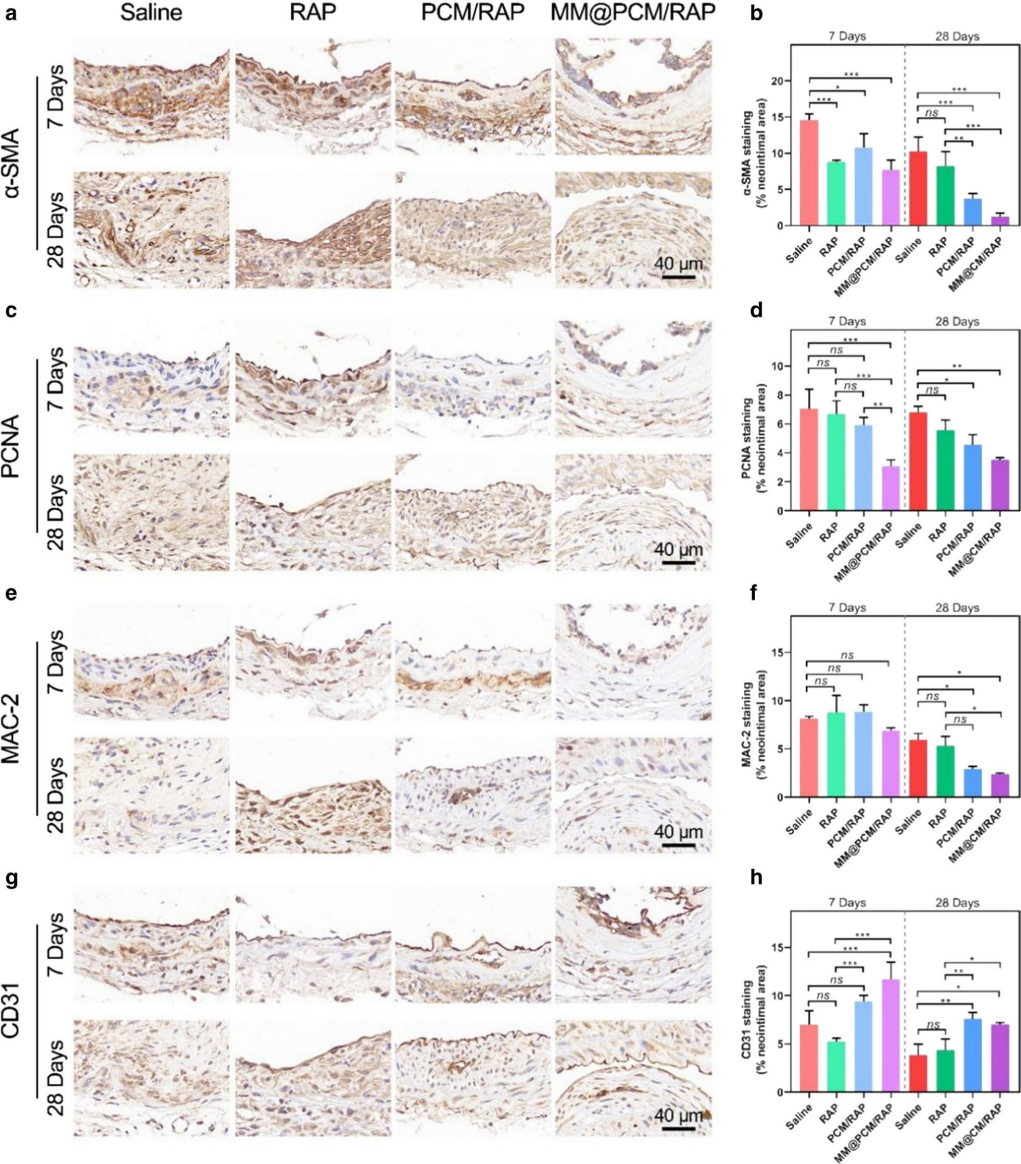
Immunohistochemistry analyses of carotid artery sections from mouse model after different treatments for 7 and 28 days. The images and quantitative analysis of carotid artery sections stained by **a**, **b** α-SMA, **c**, **d** PCNA, **e**, **f** MAC-2 and **g**, **h** CD31 (scale bar = 40 µm, *n* = 6, mean ± SD). Significances among the groups were determined by one-way ANOVA, followed by post hoc pairwise comparisons with the Tukey or Dunn honest significant difference. *p < 0.05, **p < 0.01, and ***p < 0.001. *ns*, no significance

Furthermore, immunohistochemical analysis was conducted on the mouse carotid artery section. To measure proliferation in vivo, mouse carotid artery tissues were stained with smooth muscle cell actin (α‐SMA), to identify VSMCs, and proliferating cell nuclear antigen (PCNA), to identify proliferating cells. As shown in Fig. 8a and b, after 28 days treatments, the expression of α‐SMA in the neointima of the MM@PCM/RAP group (1.3%) was significantly lower than that of the saline group (10.3%), free RAP group (8.2%) and PCM/RAP group (3.7%). Meanwhile, the PCNA staining results shown in Fig. 8c and d confirmed that the MM@PCM/RAP group had the lowest cells proliferation phase compared with other groups under the same treatment time, indicating that MM@PCM/RAP could effectively inhibit the proliferation of VSMCs, which might be resulted from the innate “homing” ability of macrophage membrane and the ROS-responsive local drug release to selectively deliver RAP to lesion efficiently and accurately.

In the pathological environment of the injured vessel, the degree of inflammation was dependent on the content of macrophages. MAC-2 (macrophage surface marker) staining was used to evaluate the degree of inflammation during treatment. As shown in Fig. 8e, the high expression of MAC-2 was observed in the media layer and the intimal layer of the blood vessel after 7 days of treatment, but the local high expression of MAC-2 disappeared after 28 days of treatment. As shown in Fig. 8e and f, with the increase of treatment time, the inflammation level of each group decreased. After 28 days of treatment, the MM@PCM/RAP group showed the lowest MAC-2 expression, which was significantly different compared with the saline group and the free RAP group, suggesting that MM@PCM/RAP could reduce the overall level of inflammation in lesion.

In addition, CD31 (endothelial cell marker) staining was used to confirm the endothelial condition of the lesion. Excessive ROS levels could cause cell damage and death by inducing lipid peroxidation. As shown in Fig. 8g and h, under the same treatment time, the expression of CD31 in the PCM/RAP group and the MM@PCM/RAP group was higher than the other two groups, implying a better endothelial compatibility, which may be due to the consumption of ROS in the inflammatory microenvironment by nanomedicines.

Collectively, a series of evidences suggested that MM@PCM/RAP exhibited excellent therapeutic effects against IH after vascular injury in mouse carotid artery injury model and showed hints of desirable treatment effects than PCM/RAP and free RAP.

### In vivo biosafety of assessment

**Fig. 9:**
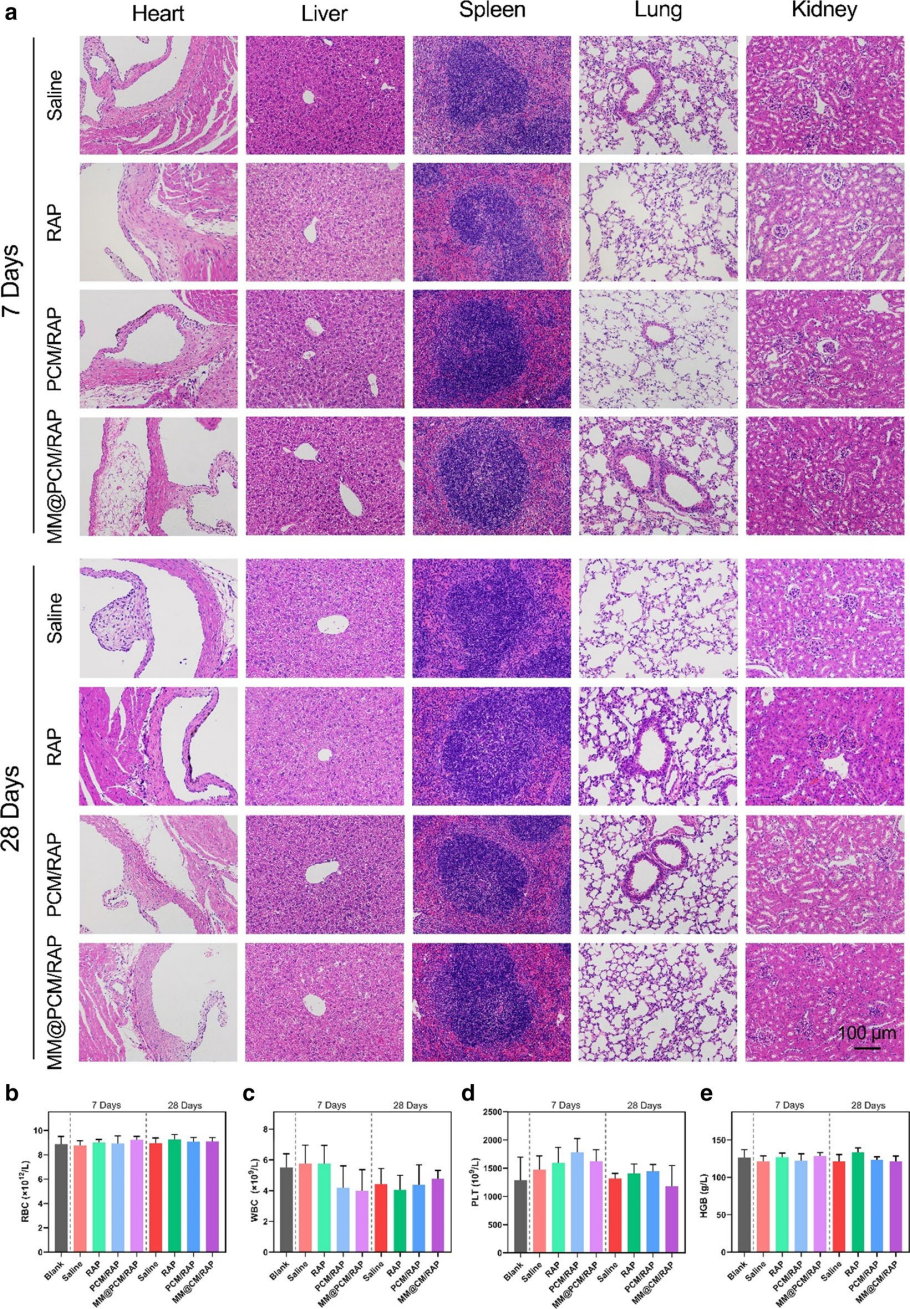
In vivo biosafety evaluation. **a** H&E staining images of main organs from mice after various treatments for 7 and 28 days (scale bar = 100 µm). **b**–**e** The RBC, WBC, PLT and HGB analysis of blood from mice after various treatments for 7 and 28 days (*n* = 6)

For the biodistribution investigation in mice, the retention content of the biomimetic nanomedicine MM@PCM/RAP was reduced in liver and spleen, revealing the reduced side effects to organism (Additional file 1: Fig. S8). The in vivo safety of biomimetic MM@PCM/RAP nanoparticles was further examined by H&E staining of the main organs of mice and blood routine analysis. The results showed that PCM/RAP and MM@PCM/RAP did not cause obvious histological toxicity to the main organs and blood toxicity, suggesting desirable biocompatibility for applications in vivo (Fig. 9a–e).

## Conclusion

In this study, engineering with the innate “homing” capacity of MM and the pathological ROS-responsive drug release characteristic of PCM, a ROS-responsive intelligent biomimetic nano-drug delivery system, MM@PCM/RAP, was developed to selectively accumulate at the injured vascular lesion and subsequently enhance the drug release in local for significantly increasing the drug bioavailability and reducing its side effects. Thus, MM@PCM/RAP could be a feasible and promising candidate for safe and efficient therapy for IH.

## Experimental section/methods

### Chemicals and reagents

Rapamycin (RAP) was purchased from Dalian Meilun Biotechnology Co. Ltd. (Dalian, China). 4-(Hydroxymethyl) phenylboronic acid pinacol ester (PBAP), 1,1-Carbonyldiimidazole (CDI) and d-mannose were acquired from Shanghai Aladdin Biochemical Technology Co., Ltd. (Shanghai, China). 4-Dimethylaminopyridine (DMAP) and deuterated dimethyl sulfoxide (DMSO-*d*_*6*_) were purchased from Shanghai Macklin Biochemical Co., Ltd. (Shanghai, China). Anhydrous dichloromethane (DCM), anhydrous dimethylformamide (DMF) and hydrogen peroxide (H_2_O_2_) 30% were acquired from Chongqing Chuandong Chemical (Group) Co., Ltd. (Chongqing, China). 1,1′-Dioctadecyl-3,3,3′,3′-tetramethylindodicarbocyanine perchlorate (DiD) was purchased from US Everbright Inc. (Suzhou, China). Membrane and cytosol protein extraction kit, 3,3′-dioctadecyloxacarbocyanine perchlorate (DiO), 4′,6-diamidino-2-phenylindole (DAPI), RIPA lysis buffer, SDS-PAGE gel quick preparation kit and polyvinylidene difluoride membrane (PVDF) were supported by Beyotime Institute of Biotechnology (Jiangsu, China). Fetal bovine serum (FBS), Dulbecco’s modified Eagle’s medium (DMEM) and Roswell Park Memorial Institute (RPMI) 1640 medium were purchased from Gibco (USA). MTS was purchased from Promega Corporation (Beijing, China). Coomassie brilliant blue and dialysis bag were purchased from Solarbio (Beijing, China). BCA Protein Assay Kit was purchased from Beyotime (Shanghai, China).

### Synthesis and characterization of ROS-scavenging carrier PCM

According the previous reports of PBAP-CDI [31, 42], PBAP (4 mmol) and CDI (8 mmol) were respectively dissolved in DCM (5 mL), and then mixed in flame-dried 50 mL round bottom flask. After immersed in an oil bath under stirring at 30 °C for 1 h, the mixture was washed with ultrapure water for 3 times (10 mL each) and physiological saline for 1 time (10 mL), followed with drying with anhydrous magnesium sulfate (MgSO_4_) for 4 h. After filtration, the white precipitate of PBAP-CDI was harvested by evaporating the solvent using a rotary evaporator under reduced pressure. Subsequently, to ensure the balance between hydrophobicity and hydrophilicity in PCM, a mixture of PBAP-CDI (1.5 mmol), *D*-mannose (0.5 mmol) and DMAP (1.5 mmol) in DMF (4 mL) was stirred overnight at 40 ℃. Then, the product was transferred to a dialysis bag (molecular weight cut-off (MWCO): 500 Da, Solarbio) and dialyzed against ultrapure water for 24 h. After the product was collected and frozen in the refrigerator at − 80 ℃, the solid pure substance of PCM was obtained by lyophilization. PBAP-CDI and PCM were dissolved in DMSO-*d*_6_ and then subjected to ^1^H NMR spectroscopic (AVANCE 500, Bruker, CH) analysis.

### Preparation of RAP-loaded ROS-responsive nanoparticle (PCM/RAP)

PCM/RAP nanoparticles were prepared though the dialysis method [32]. PCM (10 mg) and RAP (1 mg) were sufficiently dissolved in DMF (200 μL), and then the mixture was added dropwise into the high-speed rotating ultrapure water (5 mL). After dialysis in a dialysis bag (MWCO: 1000 Da) for 12 h, pure PCM/RAP nanoparticles were obtained.

### Isolation of macrophage membrane (MM)

The macrophage membrane was isolated from the murine macrophages RAW 264.7 cell line. The cells were washed three times with sterile PBS, and then lysed in buffer A (P0033, Beyotime, China) supplemented with phenylmethanesulfonyl fluoride (PMSF, ST506, Beyotime, China) in an ice bath for 10 min. The cell suspension was homogenized in a glass homogenizer for 25 times to disrupt the cell membrane. After centrifuging at 700*g*, 4 °C (Centrifuge 5418 R, Eppendorf, Germany) for 10 min, the supernatants were collected and further centrifuged at 14,000*g* for 30 min at 4 ℃. Macrophage membrane was obtained by collecting the bottom sediment.

### Preparation of biomimetic ROS-responsive nanoparticles (MM@PCM/RAP)

MM@PCM/RAP nanoparticles were obtained by an extrusion method [33]. Briefly, after washing with ultrapure water twice, the membrane was mixed with PCM/RAP, followed by ultrasonic treatment on the sonicator (FS30D, 42 kHz, 100 W) for 3 min. Then, the mixture solution was transferred into an extruder (Avestin, LF-1, Canada) and passed through a polycarbonate porous membrane (200 nm) for 16 times to harvest the MM@PCM/RAP.

### General characterization of nanoparticles

The hydrodynamic diameter (*D*_h_), distribution and zeta potential of MM, PCM/RAP and MM@PCM/RAP were performed by dynamic light scattering (DLS) using a Malvern nano-ZS Zetasizer (Nano ZS 90, Malvern, UK) with a He–Ne laser (λ = 633 nm) at a scattering angle of 90° at 25 °C. TEM samples of PCM/RAP and MM@PCM/RAP were prepared by applying one drop of the solution onto a carbon-supported copper (200 mesh) followed with 1% phosphotungstic acid staining. After air drying, the morphology of PCM/RAP and MM@PCM/RAP were observed under transmission electron microscope (TEM, JEM-2100F, JEOL, Japan).

### Drug loading efficiency and encapsulation efficiency

PCM/RAP nanoparticles were performed as previously described. The lyophilized powder of PCM/RAP was dissolved in anhydrous DMF and then the absorbance of solution was measured by UV–Vis spectrophotometer (DU730, Beckman Coulter) at 279 nm. The quality of RAP in PCM/RAP was calculated by the pre-established concentration-absorbance standard curve of RAP in DMF. Subsequently, the drug loading efficiency (*LE*) and encapsulation efficiency (*EE*) were calculated using the following equation:

1 $$LE(\% ) = \frac{{M_{RAP} }}{{M_{PCM} + M_{RAP} }} \times 100\%$$

2 $$EE(\% ) = \frac{{M_{RAP} }}{{M_{added} }} \times 100\%$$

in which *M*_*RAP*_ is the mass of RAP loaded in the nanoparticles, *M*_*PCM*_ is the mass of small molecule carrier in the formulation and *M*_*added*_ is the mass of added RAP.

### In vitro drug release of nanoparticles

The drug release profile of RAP in nanoparticles in the PBS environment without or with H_2_O_2_ (10 μM) was detected by UV–Vis spectrophotometer at 279 nm. PCM/RAP and MM@PCM/RAP solution (1 mg mL^−1^, 2 mL) were respectively added to dialysis bag (MWCO: 3500 Da, Solarbio), and then immersed in 40 mL release medium, under shaking at 500 rpm at 37 ℃ to simulate the in vivo environment. At predetermined time intervals, 2 mL of the release medium was collected and replenished with the same volume of fresh medium. The absorbance of the samples was measured and the percentage of drug released was calculated based on the pre-established standard curve.

### Co-localization study

In order to label the core of the nanoparticles, the fluorescent nanoparticles PCM/DiD were prepared by replacing RAP with a red fluorescent DiD. Then the macrophage membrane was labeled with green fluorescent DiO, and then co-extruded with PCM/DiD to obtain the red and green double fluorescent labeled nanoparticles MM/DiO@PCM/DiD. After co-incubated with MM/DiO@PCM/DiD overnight, ECs were washed with PBS followed by 4% paraformaldehyde (PFA) fixing and DAPI staining. The fluorescence of samples was observed by confocal laser scanning microscopy (CLSM, Leica, Germany) under 364, 484, and 644 nm filters.

### Characterization of proteins

The retained proteins were characterized by polyacrylamide gel electrophoresis (SDS-PAGE). The protein samples of MM@PCM/RAP, macrophage membrane (MM) and macrophage were extracted by RIPA lysis buffer contained PMSF. After adding the loading buffer, the protein samples were transferred to a metal bath at 95 °C for 10 min to fully denature the protein. Subsequently, 20 μL of each sample was loaded onto the pre-configured 10% gel. Gels were run at 140 V for 60 min to separate protein bands of different molecular weights. Bands were visualized using Coomassie brilliant blue.

The CCR2, integrin α4, integrin β1 and CD47 on the cell membrane were characterized by western blot (WB). Briefly, after separate protein bands in 8% gel, the proteins were transferred onto a PVDF membrane at 90 mA for 90 min. The PVDF membrane was blocked with 5% milk for 2 h and then incubated overnight at 4 °C with the primary antibody of CCR2 (1:1000, ab203128, Abcam), integrin α4 (1:1000, ab81280, Abcam), integrin β1 (1:1000, ab179471, Abcam) and CD47 (1:1000, ab175388, Abcam). The following day, membranes were incubated with an HRP-conjugated secondary antibody (1:5000, ab6721, Abcam) for 1 h and observed by ChemiDoc XRS imaging system (Bio-Rad, USA).

### Cell uptake by macrophages

RAW 264.7 cells were seeded at a density of 5 × 10^5^ cells/well in 24-well plates and cultured at 37 °C with 5% CO_2_ overnight. Then, 100 μg MM@PCM/DiD or PCM/DiD nanoparticles were respectively added to each well and incubated for 1 h or 3 h. After washing with PBS, the cells were fixed with 4% PFA and then stained by DAPI. Then, the fluorescent intensity of the samples was observed by CLSM. To quantify the fluorescence intensity endocytosed by cells, cells without DAPI staining was digested with trypsin and analyzed by fluorescence-activated cell sorting (FACS, BD Biosciences, CA, USA).

### Cell uptake by the activated endothelial cells

ECs were seeded at a density of 2.5 × 10^5^ cells/well in 24-well plates and cultured at 37 °C with 5% CO_2_ overnight. The activated ECs were obtained by treating the ECs with TNF-α (40 ng mL^−1^) for 24 h. Then, 100 μg MM@PCM/DiD or PCM/DiD nanoparticles were respectively incubated with the activated or non-activated ECs for 1 h or 3 h. Subsequently, samples were prepared for CLSM observation and FACS.

### Proliferation inhibition of smooth muscle cells

Vascular smooth muscle cells (VSMCs) were seeded at 2000 cells/well into 96-well plates and cultured at 37 °C with 5% CO_2_ for 24 h. Cells were starved in serum-free medium for 2 h and then incubated with different concentrations of equivalent RAP complete culture medium (from 0.5, 1, 5 to 10 μg mL^−1^) of free RAP, PCM/RAP and MM@PCM/RAP for 24 h. The cell viability was then quantified by MTS assay.

### Cell scratch assay

VSMCs were seeded in a 6-well plate and allowed to grow overnight to 100% confluence. After scratching with a 10 µL pipette tip, the cells were gently washed twice with PBS to remove the scratched cells and then starved with serum-free medium for 1 h. Subsequently, free RAP, PCM/RAP and MM@PCM/RAP were incubated with the cells in a low-serum medium (0.2% FBS) with a RAP equivalent concentration of 2.5 µg mL^−1^. The migration of cells was observed and photographed under a microscope at 0, 6, 12 and 24 h after scratching.

### In vivo toxicity of endothelial cells

ECs were seeded in a 96-well plate and allowed to grow overnight to 100% confluence. Then, the cells were cultured in serum-free medium with different concentrations of PCM or MM@PCM (from 50, 100, 250 to 500 μg mL^−1^) for 48 h. The cell viability were then quantified by MTS assay.

### In vivo developmental toxicity of zebrafish

The zebrafish AB strain was used for in vivo developmental toxicity experiment. PCM and MM@PCM nanoparticles were mixed with fish water to form a solution of equivalent PCM concentration (from 10, 50, 100, 250 to 500 µg mL^−1^). Subsequently, the solution was incubated with healthy AB wild-type zebrafish embryos at 12 hpf (12 h after fertilization), and the fresh medium was replaced every day. The survival of embryos, the growth and deformity of juvenile were observed with stereomicroscopes at 24 hpf, 48 hpf and 72 hpf.

### Hemolysis assay

Anticoagulant rabbit blood (from healthy New Zealand white rabbits) was diluted with normal saline in a volume ratio of 4:5. Then, 2 mL of ultrapure water, normal saline, PCM/RAP (1 mg mL^−1^) and MM@PCM/RAP (1 mg mL^−1^) were placed in a 37 ℃ water bath preheating for 30 min, respectively. Subsequently, 40 µL of diluted anticoagulated rabbit blood was added to each sample, and incubated in a 37 ℃ water bath for 1 h. After centrifugation at 500*g* for 5 min, the supernatant was carefully collected. The absorbance of the supernatant of each group was measured under an UV–Vis spectrophotometer at 545 nm.

### Protein adsorption of nanoparticles

Equal amounts of PCM/RAP and MM@PCM/RAP NPs solution were added to equal volumes of FBS. After vortex mixing, the samples were placed in a 37 ℃ incubator for incubation for a certain time. Subsequently, the incubated samples were centrifuged at 14,000 rpm for 15 min to precipitate the adsorbed protein nanoparticles. BCA protein detection kit was used to determine the concentration of residual protein in the supernatant, and the proportion of adsorbed protein was calculated indirectly.

### Animals

Male C57BL/6 mice (20–22 g, 7-week old) were obtained from Chongqing Medical University in Chongqing, China. All institutional and national guidelines for the care and use of laboratory animals were followed. All the animal care and experimental protocols were carried out with review and approval from the Laboratory Animal Welfare and Ethics Committee of Chongqing University (IACUC Issue No.: CQU-IACUC-RE-202109-002).

### In vivo pharmacokinetics study

To study the halflife of RBC/DiD@PLGA in circulation, 200 µL of PCM/DiD or MM@PCM/DiD was injected into the mice through the tail vein. 20 µL of blood was collected at 15, 30 min, and 1, 2, 4, 8, 12, 24, 36, 48, and 60 h after injection. The blood samples were diluted with 40 µL PBS contained 0.2 × 10^−3^ M EDTA2K in 96-well plates, and the fluorescence intensity was measured by fluorescence microplate reader (SpectraMax Gemini EM, USA).

### Establishment and treatment of mouse carotid artery injury model

Male C57BL/6 mice at 7 weeks mice were randomized into 8 groups (6 mice per group) and fed for a week. Then, carotid artery injury mouse model was performed as described previously [41]. The mice were then injected with free RAP, PCM/RAP and MM@PCM/RAP solution (200 µL) via tail vein every three days, in which each mouse was given a dose of RAP 1.2 mg kg^−1^ each time. The mice in the control group were injected with the same volume of normal saline. After 7 and 28 days of treatment, the mice were sacrificed, respectively. In addition, to examine the ex vivo target delivery, after injured the endometrium of left carotid artery, 200 µL PCM/DiD or MM@PCM/DiD was injected through the tail vein. After 24 h, mice were euthanized, perfused with PBS containing 4% paraformaldehyde and heparin sodium, and the carotid artery was isolated for imaging and fluorescence quantification using a Xenogen IVIS 200 system.

### Histology and immunohistochemistry staining of the carotid artery

The carotid arteries with various treatments were fixed with 4% paraformaldehyde (PFA) for 1 h, and then prepared to paraffin sections. For histological analysis, the sections were stained with hematoxylin–eosin (H&E). For immunohistochemistry analysis, sections were incubated with antibodies, including α-SMA (1:100, ab5694, Abcam), PCNA (1:100, ab29, Abcam), MAC-2 (1:100, ab2785, Abcam) and CD31 (1:100, ab182981, Abcam), respectively.

### In vivo biocompatibility study

For in vivo biocompatibility studies, the main organs including heart, liver, spleen, lung and kidney of mice in different groups were collected and stained with H&E to determine the toxicity. Blood was collected in heparinized whole blood test tubes and further analyzed by an animal blood cell analyzer (BC-2800vet, Mindray, China).

## Supplementary Information

**Additional file 1: Table S1.** Cell membrane camouflaged nanomedicine applied in cardiovascular diseases. **Figure S1.** ^1^H NMR spectra of PBAP-CDI and PCM. **Table S2.** Summary of RAP loading and encapsulation efficiency (*n* = 3). **Figure S2.** Hydrolysis of PCM/RAP in ultrapure water without or with 1 mM H_2_O_2_. **Figure S3.** The protein adsorbance of nanomedicine in serum. **Figure S4.** Co-location results of the dual-fluorescent nanoparticles in ECs by CLSM. Nuclei were stained with DAPI (blue), whereas nanoparticles and cell membrane were stained with DiD (red) and DiO (green), respectively. The scale bar is 10 μm. **Figure S5.** SDS-PAGE of proteins for Macrophage, MM and MM@PCM/RAP. **Figure S6.** Absorbance spectra of RAP, MM, and PCM in DMF. **Figure S7.** The standard curve of RAP in DMF. **Figure S8.** Biodistribution of nanomedicine in the main organs.

## Data Availability

All data generated or analyzed during this study are included in this published article.
