# Supplementary material for: Macrophage membrane camouflaged reactive oxygen species responsive nanomedicine for efficiently inhibiting the vascular intimal hyperplasia
Source: J Nanobiotechnology. 2021 Nov 17;19:374. doi: 10.1186/s12951-021-01119-5 (PMC8600790; doi:10.1186/s12951-021-01119-5)
Supplement: Supplementary file 1 — Additional file 1: Table S1. Cell membrane camouflaged nanomedicine applied in cardiovascular diseases. Figure S1. 1H NMR spectra of PBAP-CDI and PCM. Table S2. Summary of RAP loading and encapsulation efficiency (n = 3). Figure S2. Hydrolysis of PCM/RAP in ultrapure water without or with 1 mM H2O2. Figure S3. The protein adsorbance of nanomedicine in serum. Figure S4. Co-location results of the dual-fluorescent nanoparticles in ECs by CLSM. Nuclei were stained with DAPI (blue), whereas nanoparticles and cell membrane were stained with DiD (red) and DiO (green), respectively. The scale bar is 10 μm. Figure S5. SDS-PAGE of proteins for Macrophage, MM and MM@PCM/RAP. Figure S6. Absorbance spectra of RAP, MM, and PCM in DMF. Figure S7. The standard curve of RAP in DMF. Figure S8. Biodistribution of nanomedicine in the main organs. [file 12951_2021_1119_MOESM1_ESM.docx]

Supplementary information

Macrophage membrane camouflaged reactive oxygen species responsive nanomedicine for efficiently inhibiting the vascular intimal hyperplasia

Boyan Liu, Wenhua Yan, Li Luo, Shuai Wu, Yi Wang, Yuan Zhong, Dan Tang, Ali Maruf, Meng Yan, Kun Zhang, Xian Qin, Kai Qu, Wei Wu*, and Guixue Wang

**Table S1.** Cell membrane camouflaged nanomedicine applied in cardiovascular diseases.

| **Cell type** | **Nanoparticle** | **Indication** | **Ref.** |
| --- | --- | --- | --- |
| Red blood cell | PLGA | Inhibit the progression of atherosclerosis by prolong the blood circulation time. | [1]* |
|  | 4-(Hydroxymethyl) phenylboronic acid pinacol este conjugated 5-aminolevulinic acid prodrug | Inhibit the proliferation of macrophages and vascular smooth muscle cells *in vitro* under the stimulus of the high concentration ROS in lesion. | [2]* |
|  | “Plug and play” of DSPE-PEG-CR_8_ and DSPE-PEG-DTX | Inhibit the progression of atherosclerosis by the functional engineering of active target moiety and pH-sensitive prodrug. | [3]* |
|  | CDX peptide modification | Selectively deliver cargo into brain by the CDX peptide modification on surface of RBC | [4] |
|  | Boronic ester conjugated dextran | Reduce ischemic brain damage by prolonging the systemic circulation of NR2B9C, enhancing the active targeting of lesion | [5] |
| Macrophage | PLGA | Inhibit the progression of atherosclerosis by the active target cargo delivery. | [6]* |
|  | 4-(Hydroxymethyl) phenylboronic acid pinacol este conjugated dextran | Inhibit the proliferation of macrophages and smooth muscle cells *in vitro* by the target delivery and ROS-responsive cargo release. | [7]* |
|  | oxidation-sensitive chitosan oligosaccharide | Improve therapeutic efficacy in atherosclerosis by the synergistic effects of pharmacotherapy and inflammatory cytokines sequestration, avoiding the clearance of NPs from the reticuloendothelial system, leading NPs to the inflammatory tissues, enhancing ROS-responsiveness specific payload release, sequestering proinflammatory cytokines to suppress local inflammation. | [8] |
| Platelet | PLGA | Inhibit the coronary restenosis by the biomimetic target delivery and the local DTX release | [9] |
|  | PLGA | Image on live detection of atherosclerosis using a lipid-chelated gadolinium. | [10] |
| Endothelial cell | Copolymer consisting of *p*-Hydroxybenzyl alcohol, oxalyl chloride, and poly-(ethylene glycol) | Improve the repair of cerebral I/R injury by the enhanced target mediating by the genetic bioengineering high expression of CXCR4, the ROS responsive cargo release, and the ROS degradation of polymeric carrier for ROS scavenging. | [11]* |
| Stem cell | PLGA | Enhance the blood reperfusion in the severe hindlimb ischemia model by the overexpress CXCR4-receptor on stem cell and the local VEGF release. | [12] |
| Neural stem cell | PLGA | Augment the efficacy of glyburide, an anti-edema agent for stroke treatment by the enhanced target function from the CXCR4-overexpressing membrane-coated on surface. | [13] |
| Hybrid erythrocyte-platelet | PLGA | Exhibit long circulation and suitability for *in vivo* exploration in aortas imaging by integrating biological functions of erythrocyte and platelet. | [14] |

* The study is cited from our group.


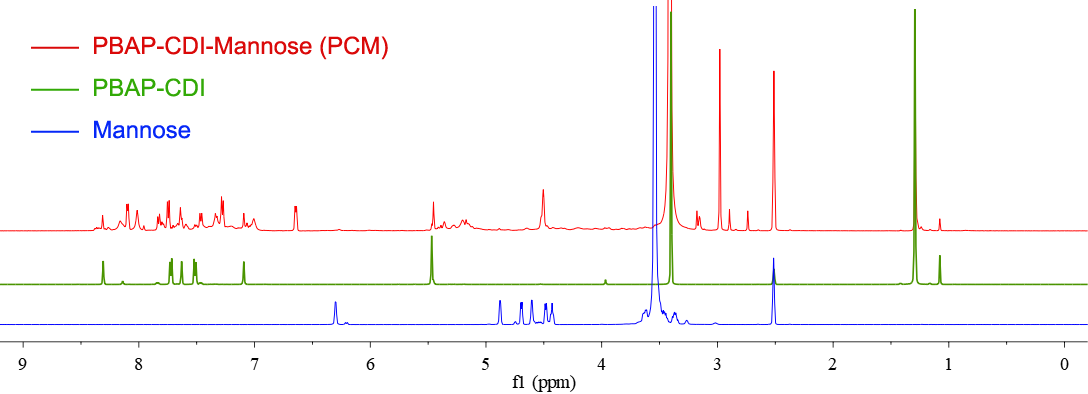


**Figure S1.** ^1^H NMR spectra of PBAP-CDI and PCM.

**Table S2.** Summary of RAP loading and encapsulation efficiency (*n*=3).

| Sample | RAP in PMR  μg mg^-1^ | Drug loading efficiency (%) | Drug encapsulation efficiency (%) |
| --- | --- | --- | --- |
| PCM/RAP | 797±2 | 7.3±0.5 | 79.7±0.2 |


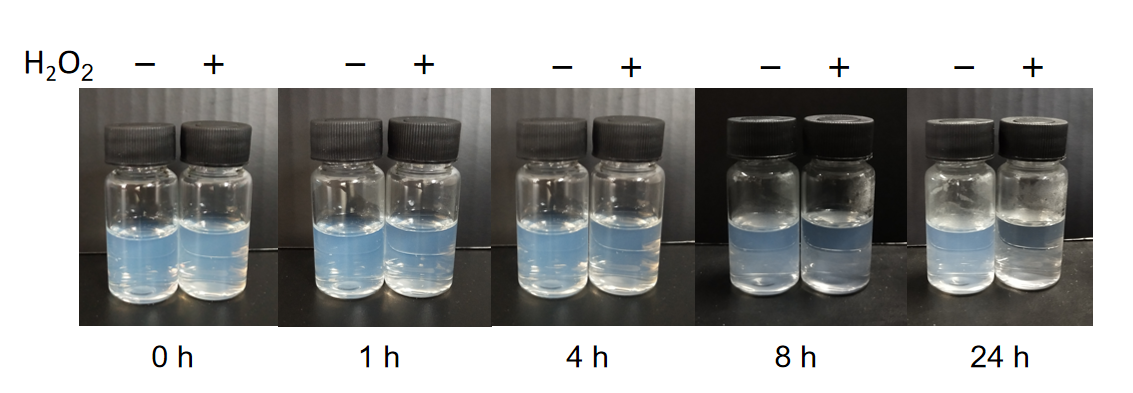


**Figure S2.** Hydrolysis of PCM/RAP in ultrapure water without or with 1 mM H_2_O_2_.


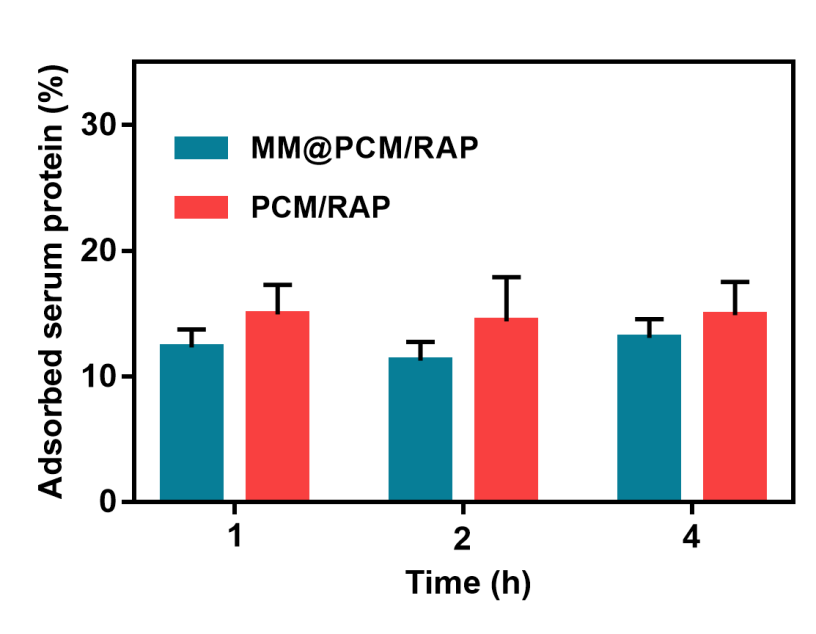


**Figure S3.** The protein adsorbance of nanomedicine in serum.


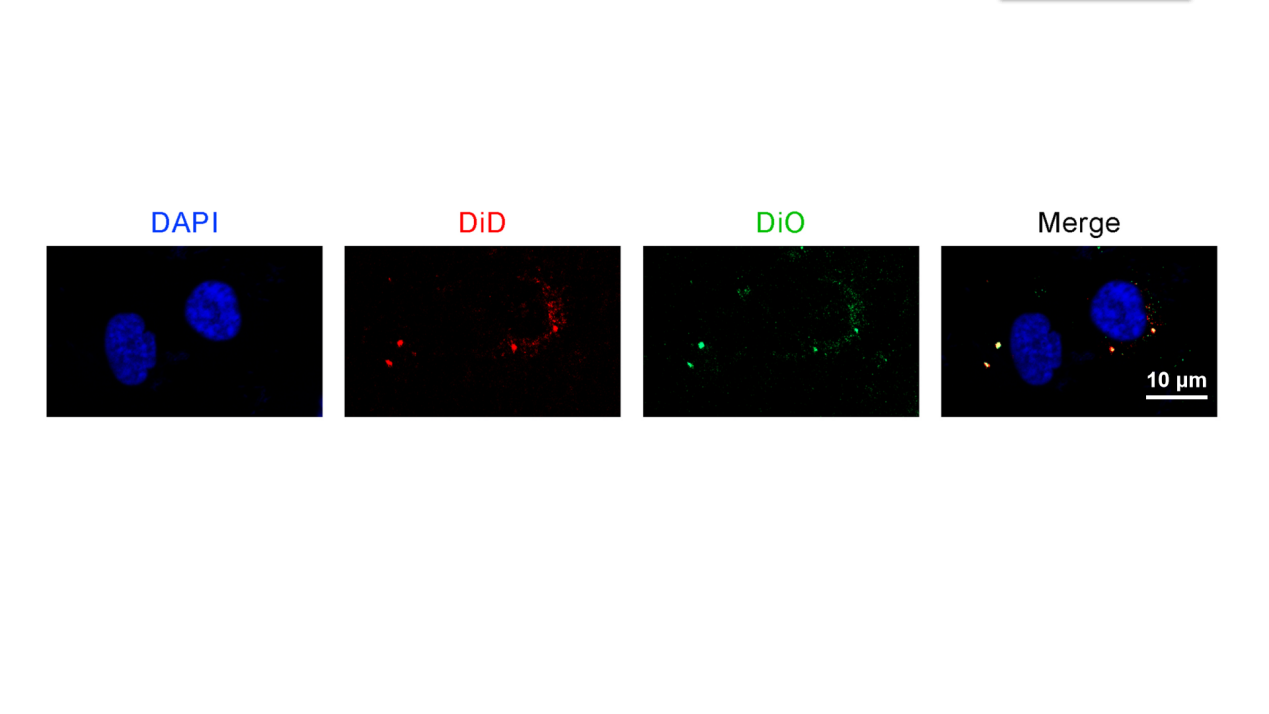


**Figure S4.** Colocation results of the dual-fluorescent nanoparticles in ECs by CLSM. Nuclei were stained with DAPI (blue), whereas nanoparticles and cell membrane were stained with DiD (red) and DiO (green), respectively. The scale bar is 10 μm.


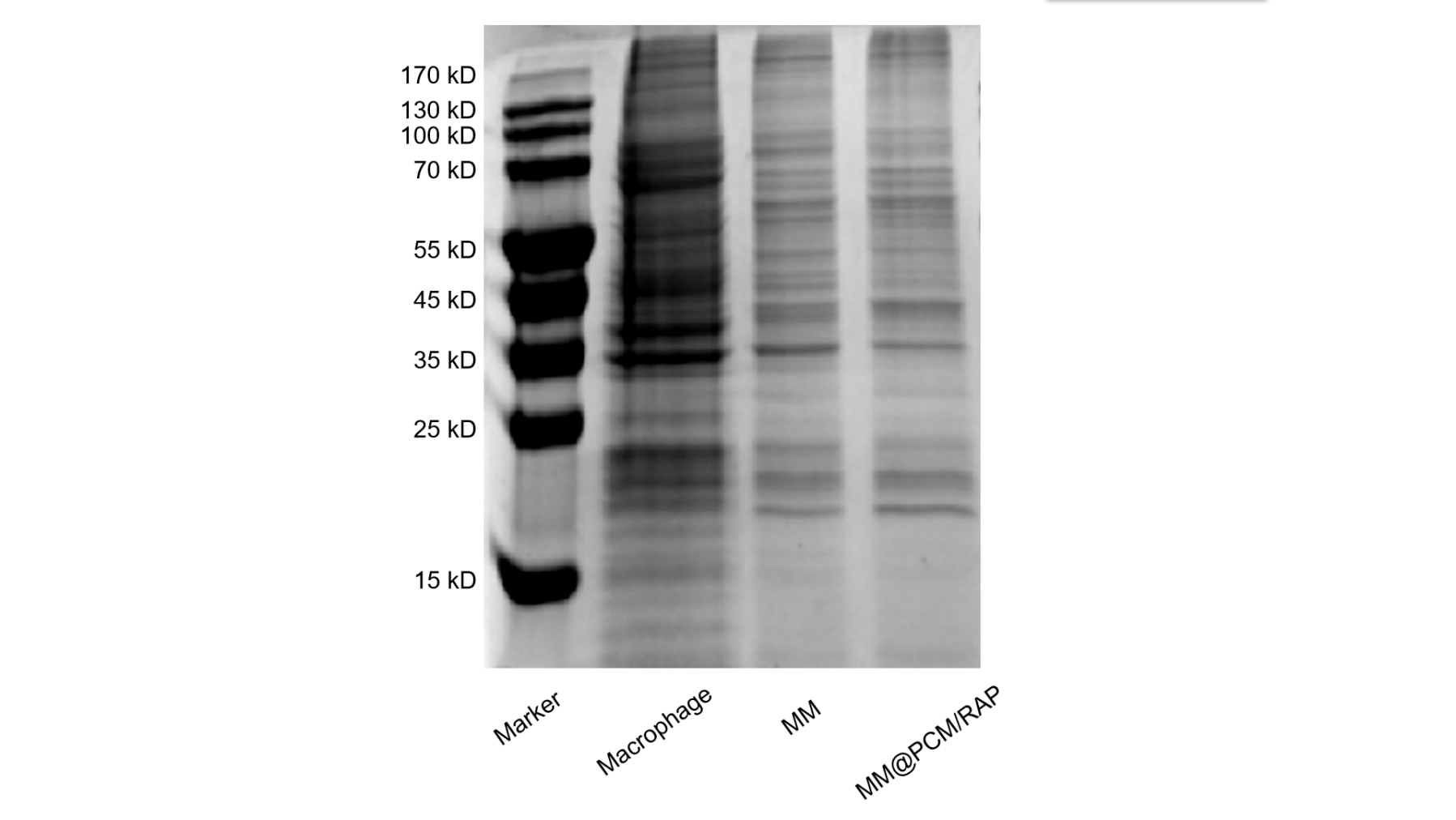


**Figure S5.** SDS-PAGE of proteins for Macrophage, MM and MM@PCM/RAP.





**Figure S6.** Absorbance spectra of RAP, MM, and PCM in DMF.


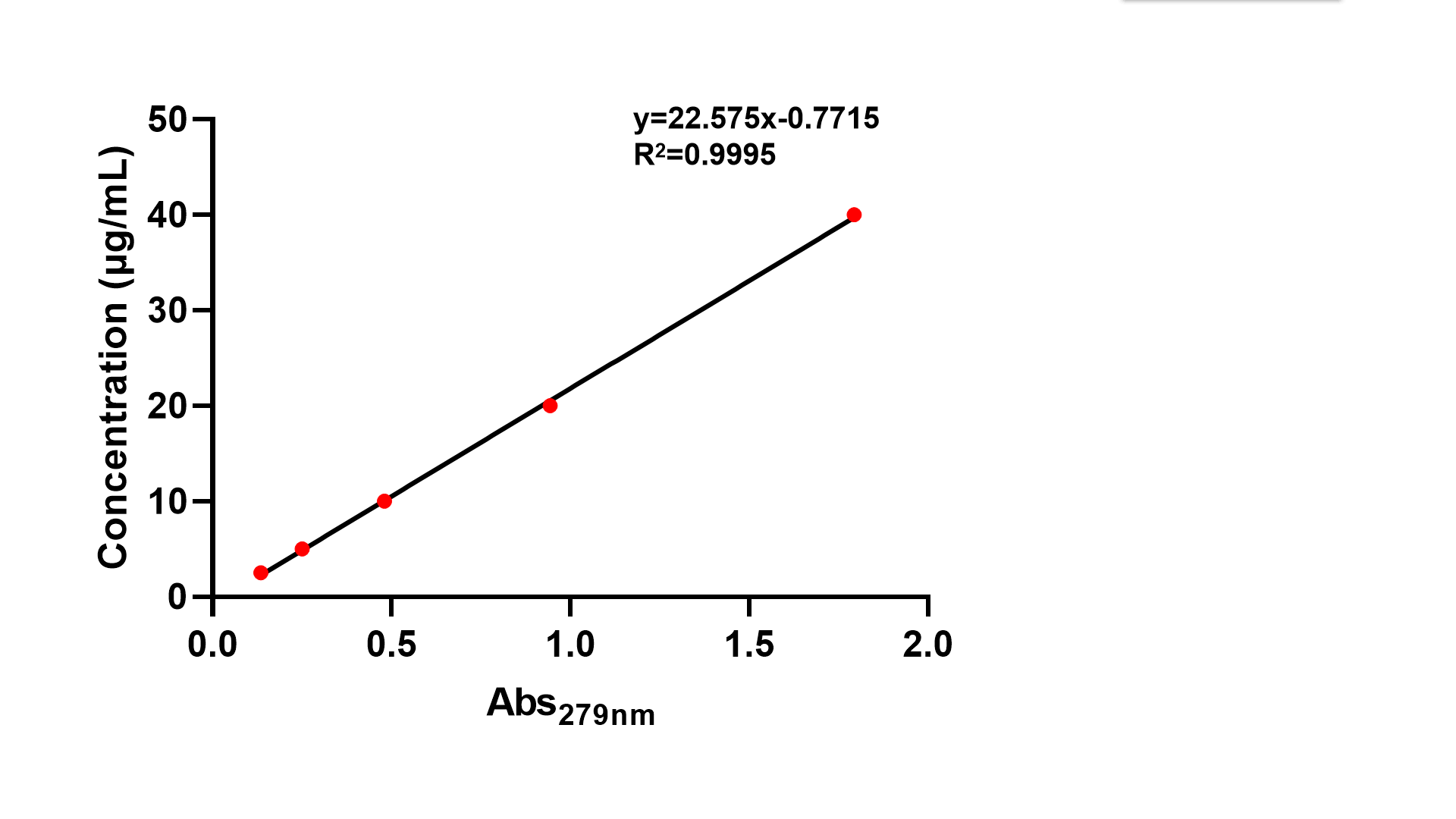


**Figure S7.** The standard curve of RAP in DMF.


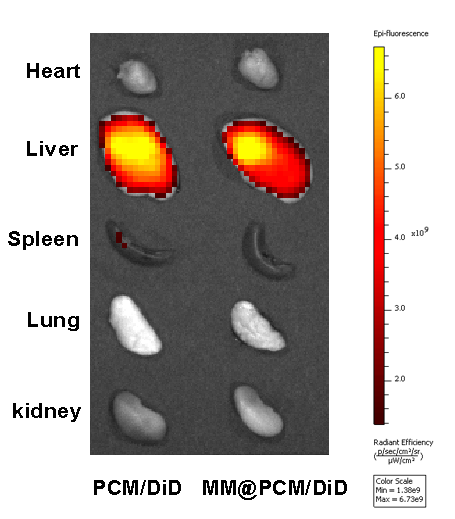


**Figure S8.** Biodistribution of nanomedicine in the main organs.

**References:**

[1] Wang Y, Zhang K, Qin X, et al. Biomimetic Nanotherapies: Red Blood Cell Based Core–Shell Structured Nanocomplexes for Atherosclerosis Management. Adv Sci. 2019;6:1900172.

[2] Maruf A, Wang Y, Luo L, et al. Nanoerythrocyte Membrane–Enveloped ROS-Responsive 5-Aminolevulinic Acid Prodrug Nanostructures with Robust Atheroprotection. Part Part Syst Char. 2020;37:2000021.

[3] Zhong Y, Qin X, Wang Y, et al. “Plug and Play” Functionalized Erythrocyte Nanoplatform for Target Atherosclerosis Management. ACS App Mater Interf. 2021;13:33862-33873.

[4] Chai Z , Hu X , Wei X , et al. A facile approach to functionalizing cell membrane-coated nanoparticles with neurotoxin-derived peptide for brain-targeted drug delivery. Journal of Controlled Release. 2017;264:102-111.

[5] Lv W, Xu J, Wang X, et al. Bioengineered Boronic Ester Modified Dextran Polymer Nanoparticles as Reactive Oxygen Species Responsive Nanocarrier for Ischemic Stroke Treatment. ACS Nano. 2018;12:5417-5426.

[6] Wang Y, Zhang K, Li T, et al. Macrophage membrane functionalized biomimetic nanoparticles for targeted anti-atherosclerosis applications. Theranostics, 2021;11:164-180.

[7] Tang D, Wang Y, Wijaya A, et al. ROS-responsive biomimetic nanoparticles for potential application in targeted anti-atherosclerosis. Regen Biomater. 2021;8:bab033.

[8] Gao C, Huang Q, Liu C, et al. Treatment of atherosclerosis by macrophage-biomimetic nanoparticles via targeted pharmacotherapy and sequestration of proinflammatory cytokines. Nat Commun. 2020;11:2622.

[9] Hu C, Fang RH, Wang KC, et al. Nanoparticle biointerfacing by platelet membrane cloaking. Nature. 2015;526:118-121.

[10] Wei X, Ying M, Dehaini D, et al. Nanoparticle Functionalization with Platelet Membrane Enables Multifactored Biological Targeting and Detection of Atherosclerosis. ACS Nano. 2018;12:109-116.

[11] Luo L, Zang G, Liu B, et al. Bioengineering CXCR4-overexpressing cell membrane functionalized ROS-responsive nanotherapeutics for targeting cerebral ischemia-reperfusion injury. Theranostics. 2021;11:8043-8056.

[12] Jc. BR, Ju KB, YoshieA, et al. Bioengineered stem cell membrane functionalized nanocarriers for therapeutic targeting of severe hindlimb ischemia. Biomaterials. 2018;185:360-370.

[13] Ma J, Zhang S, Liu J, et al. Targeted Drug Delivery to Stroke via Chemotactic Recruitment of Nanoparticles Coated with Membrane of Engineered Neural Stem Cells. Small. 2019;15:1902011.

[14] Dehaini D, Wei X , Fang R H , et al. Nanocarriers: Erythrocyte-Platelet Hybrid Membrane Coating for Enhanced Nanoparticle Functionalization. Adv Mater. 2017;29: 1606209.
